# Supplementary material for: Mineralogical control on methylotrophic methanogenesis and implications for cryptic methane cycling in marine surface sediment
Source: Nat Commun. 2022 May 17;13:2722. doi: 10.1038/s41467-022-30422-4 (PMC9114137; doi:10.1038/s41467-022-30422-4)
Supplement: Supplementary file 1 — Supplementary Information [file 41467_2022_30422_MOESM1_ESM.pdf]

Mineralogical control on methylotrophic methanogenesis and implications for cryptic methane cycling in marine surface sediment

Ke-Qing Xiao[1]*, Oliver W. Moore[1], Peyman Babakhani[1], Lisa Curti[1], Caroline L. Peacock[1]

[1]University of Leeds, School of Earth and Environment, Leeds LS2 9JT, U.K.

*Corresponding author. Email: k.q.xiao@leeds.ac.uk

Supplementary **Table 1** Isotherm parameters for methylamines adsorption.

Compound	Adsorbent	Adsorbent conc. (g L^{-1})	Partition coefficients (mL g^{-1}) K_{ads}	Partition coefficients (mL m^{-2}) K_{area}
Monomethylamine (MMA)	Chlorite	20	0.87	0.088
	Illite	20	1.61	0.082
	Kaolinite	20	1.00	0.049
	Montmorillonite	20	5.11	0.17
Dimethylamine (DMA)	Chlorite	20	1.41	0.15
	Illite	20	3.31	0.17
	Kaolinite	20	2.09	0.10
	Montmorillonite	20	6.22	0.21
Trimethylamine (TMA)	Chlorite	20	5.37	0.59
	Illite	20	7.60	0.39
	Kaolinite	20	5.91	0.29
	Montmorillonite	20	18.57	0.63

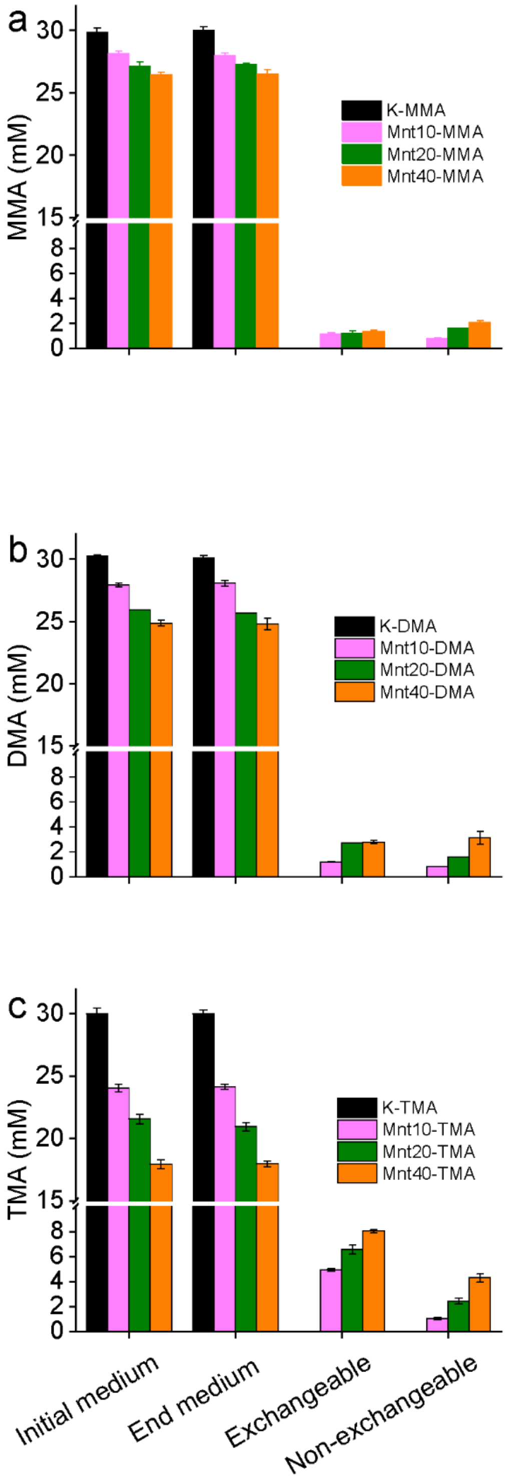

Supplementary Fig. 1 Distribution of monomethylamine (MMA) (a), dimethylamine (DMA) (b) and trimethylamine (TMA) (c) in different pools in abiotic control experiments, in the absence (K) and presence of 10 (Mnt10), 20 (Mnt20) and 40 (Mnt40) g L^{-1} montmorillonite.

Free methylamines in initial medium (after 24 hours equilibrium) (initial medium), free methylamines in medium after 144 hours (end medium), exchangeable methylamines adsorbed by montmorillonite (after 144 hours) extracted using 1 M LiCl (exchangeable pool) and non-exchangeable methylamines adsorbed by montmorillonite (after 144 days) extracted using 5 M HF-1 M HCl (non-exchangeable pool, values are transformed into concentrations related to initial medium volume 10 mL for comparison). Data points are presented as averages and standard deviation of triplicate samples.

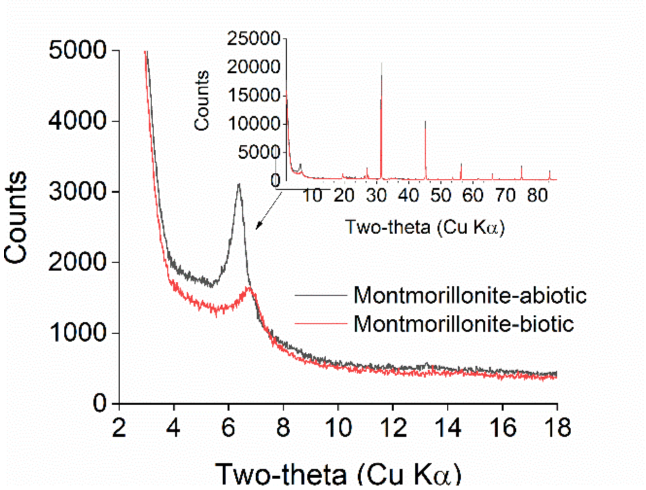

Supplementary Fig. 2 X-ray diffraction patterns of montmorillonite (SWy-3) before (abiotic) and after 144 days bioreduction (biotic), showing that the 001 peak shifts to lower d-spacing (higher two-theta) and becomes broader and less intense.

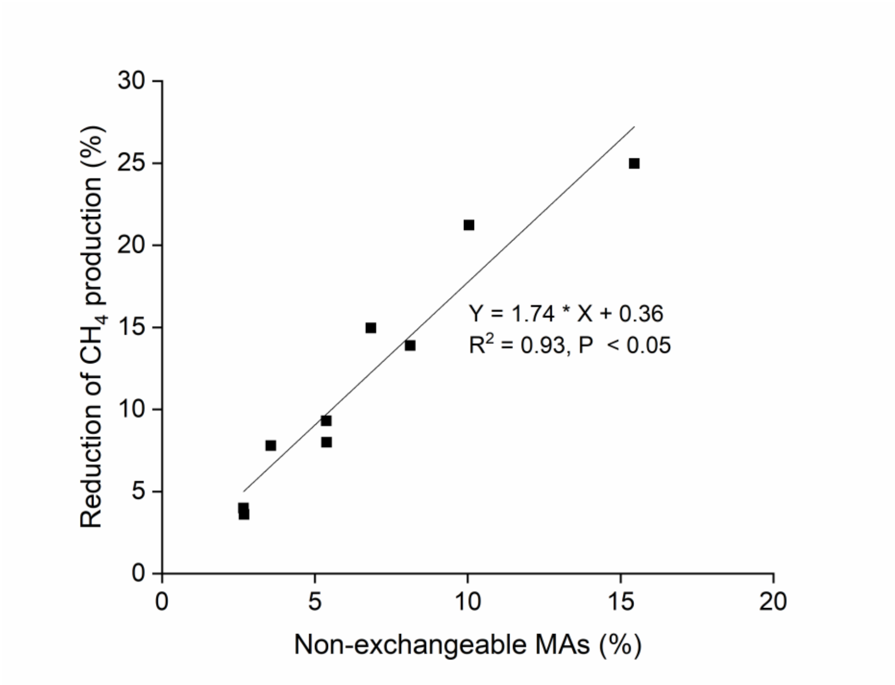

Supplementary Fig. 3 Correlation between non-exchangeable MAs (%) and reduction in final methane production (%).

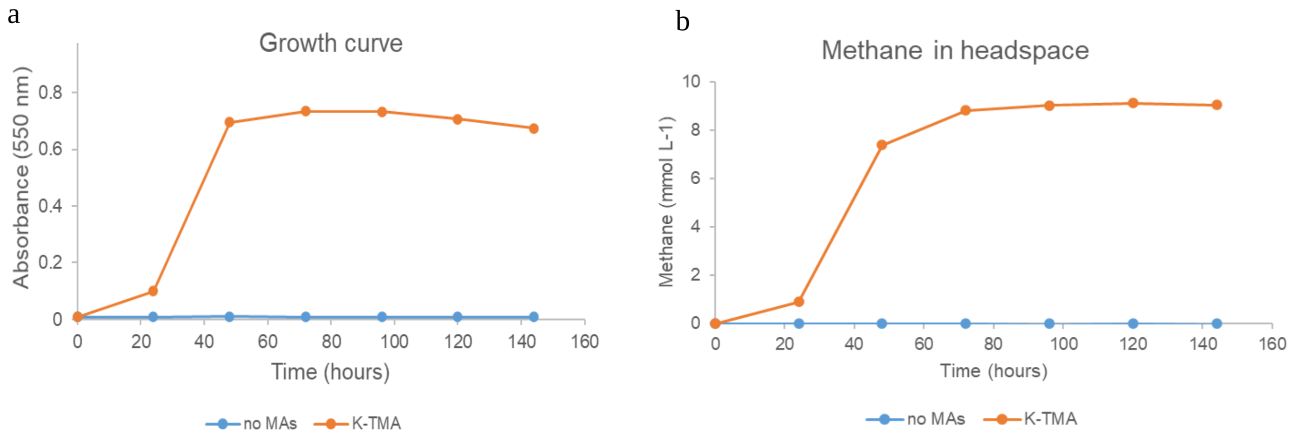

Supplementary Fig. 4 Growth curve (OD_{550nm}) of *Methanococcoides methylutens TMA-10* (a) and methane production in medium without methylamines (MAs) compared to the presence of trimethylamine (TMA) (b).
