# Supplementary material for: Mineralogical control on methylotrophic methanogenesis and implications for cryptic methane cycling in marine surface sediment
Source: Nat Commun. 2022 May 17;13:2722. doi: 10.1038/s41467-022-30422-4 (PMC9114137; doi:10.1038/s41467-022-30422-4)
Supplement: Supplementary file 3 — Reporting Summary [file 41467_2022_30422_MOESM3_ESM.pdf]

nature portfolio

Corresponding author(s): Ke-Qing Xiao

Last updated by author(s): Apr 11, 2022

Reporting Summary

Nature Portfolio wishes to improve the reproducibility of the work that we publish. This form provides structure for consistency and transparency in reporting. For further information on Nature Portfolio policies, see our Editorial Policies and the Editorial Policy Checklist.

Statistics

For all statistical analyses, confirm that the following items are present in the figure legend, table legend, main text, or Methods section.

n/a	Confirmed	
☐	☒	The exact sample size (*n*) for each experimental group/condition, given as a discrete number and unit of measurement
☐	☒	A statement on whether measurements were taken from distinct samples or whether the same sample was measured repeatedly
☐	☒	The statistical test(s) used AND whether they are one- or two-sided<br>*Only common tests should be described solely by name; describe more complex techniques in the Methods section.*
☐	☒	A description of all covariates tested
☒	☐	A description of any assumptions or corrections, such as tests of normality and adjustment for multiple comparisons
☐	☒	A full description of the statistical parameters including central tendency (e.g. means) or other basic estimates (e.g. regression coefficient) AND variation (e.g. standard deviation) or associated estimates of uncertainty (e.g. confidence intervals)
☐	☒	For null hypothesis testing, the test statistic (e.g. *F*, *t*, *r*) with confidence intervals, effect sizes, degrees of freedom and *P* value noted<br>*Give P values as exact values whenever suitable.*
☒	☐	For Bayesian analysis, information on the choice of priors and Markov chain Monte Carlo settings
☒	☐	For hierarchical and complex designs, identification of the appropriate level for tests and full reporting of outcomes
☒	☐	Estimates of effect sizes (e.g. Cohen's *d*, Pearson's *r*), indicating how they were calculated

Our web collection on statistics for biologists contains articles on many of the points above.

Software and code

Policy information about availability of computer code

Data collection	Agilent 7890a Gas Chromatography System,ThermoScientific ICS5000 Ion Chromatography System, Bruker D8 XRD , Axis2000 software
Data analysis	All analyses were performed using Microsoft Office 2018 and SPSS 18.0

For manuscripts utilizing custom algorithms or software that are central to the research but not yet described in published literature, software must be made available to editors and reviewers. We strongly encourage code deposition in a community repository (e.g. GitHub). See the Nature Portfolio guidelines for submitting code & software for further information.

Data

Policy information about availability of data

All manuscripts must include a data availability statement. This statement should provide the following information, where applicable:

- Accession codes, unique identifiers, or web links for publicly available datasets
- A description of any restrictions on data availability
- For clinical datasets or third party data, please ensure that the statement adheres to our policy

All data needed to evaluate the conclusions in the paper are present in the main manuscript and/or the Supplementary Materials. Source data directly used to make figures was deposited in a suitable on line repository. The data in the main manuscript, Supplementary Materials and in the on line repository will represent the minimal dataset necessary to interpret, replicate and build upon the methods or findings reported in the manuscript.

Field-specific reporting

Please select the one below that is the best fit for your research. If you are not sure, read the appropriate sections before making your selection.

- [ ] Life sciences
- [ ] Behavioural & social sciences
- [x] Ecological, evolutionary & environmental sciences

For a reference copy of the document with all sections, see nature.com/documents/nr-reporting-summary-flat.pdf

Ecological, evolutionary & environmental sciences study design

All studies must disclose on these points even when the disclosure is negative.

Study description	This study was conducted to investigate the link between mineral adsorption and microbial remineralisation during interactions between methylamines (MAs), methanogens and clay minerals in marine sediment. Three methylamines (methylamine (MMA), dimethylamine (DMA) and trimethylamine (TMA)) and four common clay minerals in marine sediment (chlorite, illite, kaolinite and montmorillonite) were chosen for experiments, and Methanococcoides methylutens TMA-10 (M. methylutens for abbreviation) was used as a representative methylotrophic methanogen. We chose n = 3 for all experiments.
Research sample	Four common clay minerals in marine sediment (montmorillonite, chlorite, illite and kaolinite) are chosen for experiments, ordered from the Clay Minerals Society . Methanococcoides methylutens TMA-10 (ordered from Deutsche Sammlung von Mikroorganismen und Zellkulturen GmbH, no.DSMZ 2657) is used as a representative methylotrophic methanogen. M. methylutens is mesophilic and originally isolated from marine sediment, and methanogens belonging to this genus are ubiquitously detected and often dominant in marine surface sediment.
Sampling strategy	Sampling was done on a daily basis, and we chose n = 3 for all experiments in order to have the minimal number of replicates for a robust statistical analysis.
Data collection	Ke-Qing Xiao collected all data for basic analysis, including methane, pH, XRD, Fe concentrations, etc.<br>Oliver W. Moore, Lisa Curti, Caroline L. Peacock collected data for scanning transmission X-ray microscopy near edge X-ray absorption fine structure spectroscopy on Beamline I08, Diamond Light Source Ltd, Oxfordshire, UK.
Timing and spatial scale	This experiment included multiple experiments and analyses spanning two years from 2019.06.19 to 2021.03.31. After proof of concept work, experiments were sampled on a daily basis to capture the full behavior of the investigated systems.
Data exclusions	No data were excluded.
Reproducibility	All attempts to repeat the experiments were successful. All data was repeated in triplicate (n=3) and uncertainties are provided on each figure.
Randomization	Not relevant as the same people took responsibility for a whole experimental and analysis process.
Blinding	Blinding is not relevant here. I don't have any other participant and all data analysis was done by the same people who had control of the whole experiment.

Did the study involve field work? [ ] Yes [x] No

Reporting for specific materials, systems and methods

We require information from authors about some types of materials, experimental systems and methods used in many studies. Here, indicate whether each material, system or method listed is relevant to your study. If you are not sure if a list item applies to your research, read the appropriate section before selecting a response.

Materials & experimental systems

n/a	Involved in the study	
[x]	[ ]	Antibodies
[x]	[ ]	Eukaryotic cell lines
[x]	[ ]	Palaeontology and archaeology
[ ]	[x]	Animals and other organisms
[x]	[ ]	Human research participants
[x]	[ ]	Clinical data
[x]	[ ]	Dual use research of concern

Methods

n/a	Involved in the study	
[x]	[ ]	ChIP-seq
[x]	[ ]	Flow cytometry
[x]	[ ]	MRI-based neuroimaging

Animals and other organisms

Policy information about studies involving animals; ARRIVE guidelines recommended for reporting animal research

Laboratory animals	Methanogenic archaea-Methanococcoides methylutens TMA-10 was ordered from Deutsche Sammlung von Mikroorganismen und Zellkulturen GmbH, no.DSMZ 2657.
Wild animals	Not applicable.
Field-collected samples	Not applicable.
Ethics oversight	Material Transfer Agreement between DSMZ GmbH and the receiving customer was signed and strictly obeyed, see attacted signed form.

Note that full information on the approval of the study protocol must also be provided in the manuscript.
